# Regulation of Proteins in Human Skeletal Muscle: The Role of Transcription

**DOI:** 10.1038/s41598-020-60578-2

**Published:** 2020-02-26

**Authors:** Pavel A. Makhnovskii, Victor G. Zgoda, Roman O. Bokov, Elena I. Shagimardanova, Guzel R. Gazizova, Oleg A. Gusev, Evgeny A. Lysenko, Fedor A. Kolpakov, Olga L. Vinogradova, Daniil V. Popov

**Affiliations:** 1 Institute of Biomedical Problems of the Russian Academy of Sciences, Moscow, 123007 Russia; 2 V.N. Orekhovich Research Institute of Biomedical Chemistry, Moscow, 119121 Russia; 3 Institute of Fundamental Medicine and Biology, Kazan (Volga Region) Federal University, Kazan, 420012 Russia; 4 KFU-RIKEN Translational Genomics Unit, Cluster for Science, Technology and Innovation Hub, RIKEN, Saitama, 351-0198 Japan; 5 RIKEN Preventive Medicine & Diagnosis Innovation Program, RIKEN, Yokohama, 230-0045 Japan; 6 RIKEN Center for Integrative Medical Sciences, RIKEN, Yokohama, 230-0045 Japan; 7 Faculty of Fundamental Medicine, M.V. Lomonosov Moscow State University, Moscow, 119991 Russia; 8 Institute of Computational Technologies of the Siberian Branch of the Russian Academy of Sciences, Novosibirsk, 630090 Russia

**Keywords:** Proteomics, Transcriptomics, Metabolism

## Abstract

Regular low intensity aerobic exercise (aerobic training) provides effective protection against various metabolic disorders. Here, the roles played by transient transcriptome responses to acute exercise and by changes in baseline gene expression during up-regulation of protein content in human skeletal muscle were investigated after 2 months of aerobic training. Seven untrained males were involved in a 2 month aerobic cycling training program. Mass-spectrometry and RNA sequencing were used to evaluate proteome and transcriptome responses to training and acute exercise. We found that proteins with different functions are regulated differently at the transcriptional level; for example, a training-induced increase in the content of extracellular matrix-related proteins is regulated at the transcriptional level, while an increase in the content of mitochondrial proteins is not. An increase in the skeletal muscle content of several proteins (including mitochondrial proteins) was associated with increased protein stability, which is related to a chaperone-dependent mechanism and/or reduced regulation by proteolysis. These findings increase our understanding of the molecular mechanisms underlying regulation of protein expression in human skeletal muscle subjected to repeated stress (long term aerobic training) and may provide an opportunity to control the expression of specific proteins (e.g., extracellular matrix-related proteins, mitochondrial proteins) through physiological and/or pharmacological approaches.

## Introduction

Metabolic syndrome and type 2 diabetes mellitus are among the most widespread diseases in Western countries. Regular low intensity aerobic exercise (aerobic training) improves insulin sensitivity, fat metabolism, and endurance. This effect is associated mainly with a marked increase in skeletal muscle mitochondrial volume/density, increased amounts and activity of mitochondrial enzymes, and increased fat oxidation in muscle both at rest and during low intensity exercise^1–4^. Therefore, investigation of the molecular mechanisms underlying skeletal muscle adaptation to regular aerobic exercise (aerobic training) is of fundamental importance.

Acute stress (e.g., each individual exercise session) is associated with transient (over several hours) changes in gene expression in cells; hence, repeated exposure to stress leads to an increase in expression of specific proteins and cellular functions^5,6^. However, regular aerobic exercise is associated not only with a transient transcriptome response after each exercise session, but also with a pronounced change in expression of a large number of genes under baseline conditions^7–9^. Therefore, both repeated responses to acute exercise and changes in baseline gene expression might regulate the tissue content of different proteins. Recent studies examined the role of baseline mRNA levels in regulating proteins in cells [see the review^6^] and, to a lesser extent, in various human tissues^10–12^. However, to the best of our knowledge, only one study has examined the role of mRNA in protein regulation in human skeletal muscle after repeated stress (i.e., at baseline before and after long term aerobic exercise training)^13^. The study demonstrated a lack of direct relation between transcriptional and proteomic abundances after long term aerobic exercise training.

**Figure 1:**
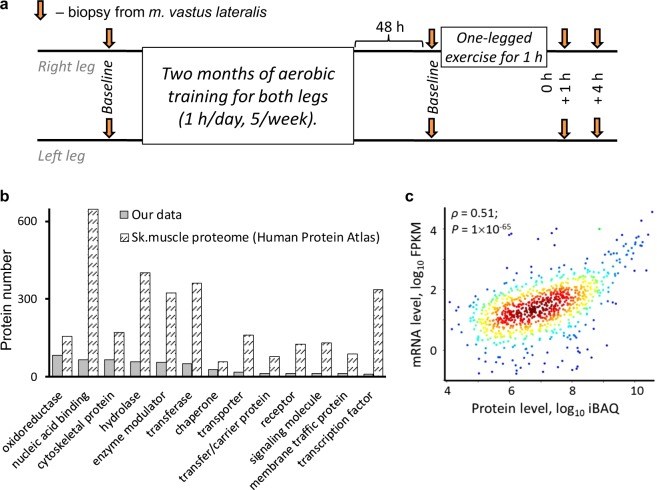
Expression of highly abundant human skeletal muscle proteins is regulated weakly at the mRNA level. **(a**) Changes in protein expression in both vastus lateralis muscles of seven untrained males were examined after 2 months of aerobic training on a cycling ergometer using HPLC-MS/MS with isobaric labeling (iTRAQ). After 2 months, changes in baseline expression of the corresponding genes, as well as contractile activity-specific mRNA responses to acute exercise (differences in gene expression between exercised and non-exercised [control] muscles at 1 and 4 h after a one-legged aerobic exercise) were examined by RNA sequencing. The design of the study provided an opportunity to eliminate effects associated with circadian regulation and systemic factors and to identify contractile activity-specific transcriptome response. **(b**) Depth of proteomic data for different protein classes (Panther Protein Class) relative to the human skeletal muscle proteome (The Human Protein Atlas). (**c**) Spearman’s correlation analysis of baseline protein expression and expression of corresponding mRNAs in untrained muscle. Each point represents the median value. Colors denote density.

The purpose of the present study was to evaluate the roles of transient transcriptome responses to acute aerobic exercise, and of changes in baseline gene expression after regular exercise training in regulating the protein content of human skeletal muscle. We examined changes in basal protein expression (HPLC-MS/MS with isobaric labeling) and corresponding mRNA (RNA sequencing) in the vastus lateralis muscle of both legs after 2 months of aerobic cycling training (1 h/day for 5 weeks) in seven untrained males. After the training program, contractile activity-specific transcriptome responses to acute aerobic exercise (one-legged knee extensions for 1 h) were evaluated. For this, tissue samples were taken from exercised and non-exercised contralateral (control) muscle, and differences in gene expression between muscles at 1 and 4 h after cessation of exercise were examined (i.e., contractile-activity-specific transcriptome responses; Fig. 1a), as described elsewhere^7^.

## Results and Discussion

Changes in the protein content of human skeletal muscle after regular exercise (2 months of aerobic exercise training) were compared with changes in baseline transcriptome profiles and with contractile activity-specific transcriptome responses to acute exercise. Previously, we showed that this training program increased ADP-stimulated mitochondrial respiration in permeabilized muscle fibers, and endurance performance in an incremental one-legged knee extension exercise and an incremental cycling test^7,14^.

### Absolute mRNA levels have little effect on the content of highly abundant proteins

In total, we identified 795 muscle proteins before and after 2 months of aerobic exercise training (Dataset 1). This covers 14% of the confirmed human skeletal muscle proteome (5749 proteins from the Human Protein Atlas with a reliability score of “Enhanced”, “Supported”, or “Approved”). The largest groups of proteins identified in our proteomic analysis were “*Oxidoreductases*”, “*Nucleic acid binding*” (predominantly RNA binding) proteins, and “*Cytoskeletal proteins*”, while the deepest coverage (40–55%) was found for “*Oxidoreductases*”, “*Cytoskeletal proteins*”, and “*Chaperones*” (Fig. 1b). Only a small number of regulatory proteins (*Transcription factors*, *Signaling molecules*, *Receptors*, and *Transporters*) were identified (Fig. 1b). Thus, our analysis identified mainly highly abundant proteins (in terms of mass concentration).

We performed Spearman’s correlation analysis to examine how strongly mRNA levels affect protein levels. We found a weak correlation (ρ = 0.51, R^2^ = 0.26, *P* < 0.001) between these parameters (Fig. 1c), which is in agreement with previous studies of various human tissues^10–12^. The wide variation in the protein:mRNA expression ratio suggests that protein expression is weakly regulated at the mRNA level and supports the notion that using mRNA levels to predict protein expression is not valid^15^. Another explanation for the huge disparity is that some proteins are poorly regulated at the mRNA level whereas others are strongly dependent on mRNA. Moreover, we suggest that this characteristic depends on the function of the protein. This hypothesis is in line with studies demonstrating that mRNAs and proteins with different functions show differing stability^16,17^. To identify proteins that are regulated at the mRNA level, we compared changes in protein levels with changes in mRNA levels after 2 months of aerobic training.

### Highly abundant muscle proteins with different functions are differentially regulated at the transcriptional level

**Figure 2:**
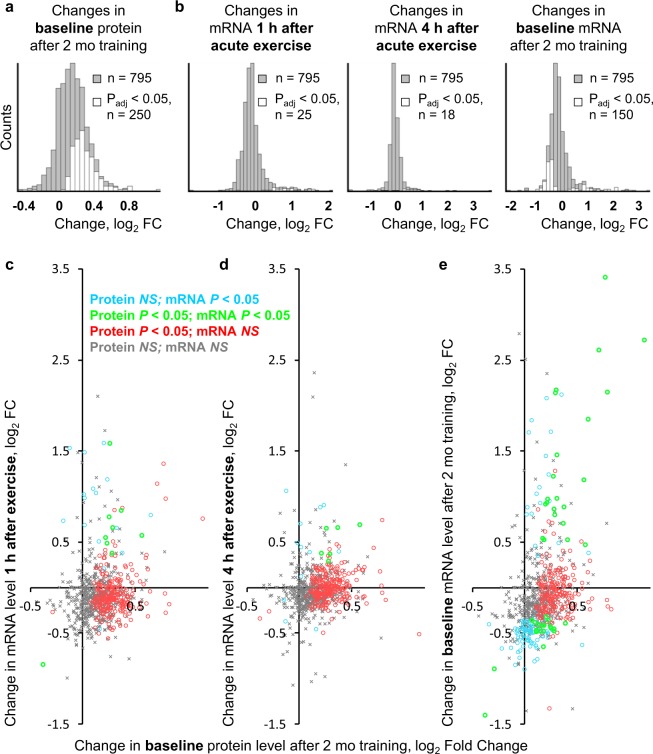
Training-induced increases in expression of highly abundant proteins in human skeletal muscle are regulated partially by baseline changes in mRNA levels. **(a**,**b**) Distribution of changes in baseline protein levels after aerobic training (a), and changes in mRNA levels at 1 and 4 h after acute aerobic exercise and at baseline after the training period (**b**). (**c**–**e**) Comparison of changes in baseline protein levels with changes in mRNA levels after acute aerobic exercise (**c**,**d**) and at baseline after the training period (**e**) identified groups of proteins showing different patterns of regulation at the transcriptional level. Different colors denote different regulation patterns. Each point represents a median value.

**Figure 3:**
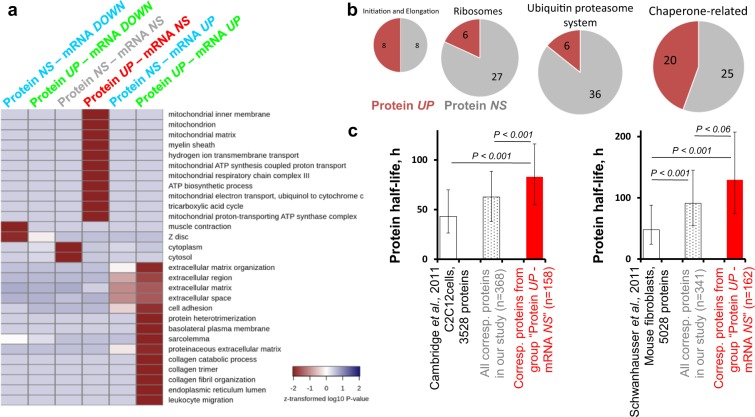
The pattern of protein regulation during adaptation to regular endurance exercise depends strongly on protein function. **(a**) Functional enrichment analysis (Gene Ontology BP and CC) revealed that the pattern of protein regulation (protein expression at baseline *vs*. mRNA expression at baseline) depends strongly on protein function. **(b**) The number and fraction of upregulated proteins, belonging to various cellular systems regulating proteostasis, after the training period. **(c**) The mean half-life of several thousand proteins (data from two cellular studies), the corresponding proteins belonging to the group “*Protein UP–mRNA NS*”, and all other corresponding proteins identified in the present study (Dataset 2). Data are expressed as the median and interquartile range.

Adaptation to regular aerobic exercise is associated not only with a transient (over several hours) increase in expression of many genes after exercise, but also with a pronounced change in expression of a large number of other genes under baseline conditions^7,13^. Therefore, we compared baseline changes in protein expression induced by 2 months of regular aerobic exercise with changes in mRNA levels after acute exercise and with baseline changes in mRNA levels after 2 months of training. Long term training predominantly increased baseline expression of proteins [248 were upregulated, median and interquartile range: log_2_ Fold change 0.28(0.20–0.37), and only two were downregulated, log_2_ Fold change −0.29 and −0.38]. The pattern of mRNA responses under all experimental conditions was quite different from that of protein responses (Fig. 2a,b). Namely, an increase in baseline protein expression was not associated with changes in expression of the corresponding mRNA at 1 and 4 h post-acute exercise (Fig. 2c,d), whereas it was associated with either an increase [n = 41, log_2_ Fold change 0.96(0.79–1.71)], no change, or even a decrease [n = 109, log_2_ Fold change −0.48(−0.56– −0.42)] in baseline mRNA expression (Fig. 2e) that is in line with the previous discussion^18^. Remarkably, functional enrichment analysis revealed that the pattern of protein regulation depends on protein function. The group “*Protein UP–mRNA UP*” was associated with extracellular matrix and collagen fibril organization, and the group “*Protein UP–mRNA NS (non-significant)*” was associated with mitochondrion and mitochondrial ATP synthesis. The group “*Protein UP–mRNA DOWN*” showed no enrichment. Other groups showing enrichment (“*Protein NS–mRNA DOWN*” and “*Protein NS–mRNA NS*”) were associated with sarcomere-related and cytosolic proteins, respectively (Fig. 3a, Dataset 2). Collectively, these data suggest that highly abundant skeletal muscle proteins with different functions show differential regulation at the transcriptional level; for example, an increase in the content of extracellular matrix-related proteins is regulated at the transcriptional level, while an increase in the content of mitochondrial proteins is not. These findings raise important questions: How are mitochondrial and some other proteins upregulated without changes in mRNA level? And what are the potential mechanisms that determine the pattern of protein regulation?

### Regular exercise up-regulates chaperone-related proteins

Surprisingly, we found that a significant number (n = 209) of some proteins (including mitochondrial proteins, n = 109) were not regulated at the transcriptional level, either at baseline after long term training or acute exercise. This is in agreement with a study showing that increased expression of many mitochondrial proteins in HeLa cells subjected to misfolding stress occurs without increased expression of the corresponding genes^19^. The amounts of individual proteins might be controlled by different post-transcriptional mechanisms, including protein synthesis on ribosomes, protein degradation (predominantly via the ubiquitin proteasome system), and regulation of protein stability. Here, we found several dozen proteins related to the ubiquitin proteasome system, ribosomes, or chaperones. In contrast to other groups, we found that half of detected chaperone and chaperone-related proteins were upregulated (20 of 45) at baseline after 2 months of training (Fig. 3b, Dataset 2). Importantly, those upregulated proteins included three mitochondrial chaperones. Rodent studies that drive or suppress gene expression show that a chaperone and chaperon-related protein (HS71A, HSF1) strongly upregulate mitochondrial density and oxidative metabolism in skeletal muscle, along with insulin sensitivity and endurance^20,21^. During the first hours of recovery after acute aerobic exercise, dozens of enzymes involved in energy metabolism are downregulated^22^. Therefore, chaperone-related proteins seem to play a meaningful role in mitochondrial biogenesis by improving mitochondrial proteostasis. In line with this, we revealed (Fig. 3c) that proteins in the group “*Protein UP–mRNA NS*” were significantly more stable (by ∼32–42%) than all other proteins identified in our study, and were more stable (by ∼92–170%) than proteins in proteomics datasets from two previous studies investigating the baseline half-life of several thousand proteins in C2C12 and mouse fibroblasts^16,23^. Notably, the use of data from C2C12 and fibroblasts studies gave actually the equal results (Fig. 3c).

Mitochondria play a central role in ATP production; therefore, mitochondrial biogenesis is an essential process for the vast majority of cells. A given -fold increase in expression of highly abundant proteins (including mitochondrial proteins) involves a significantly larger number of molecules than equivalent -fold increase in expression of low abundancy proteins; hence, the total energy cost of synthesizing highly abundant proteins is much greater than that of low abundancy proteins. This explains why, in terms of energy cost, transcriptional regulation of highly abundant proteins is less effective than post-transcriptional regulation^6^. Indeed, mRNA levels and transcription rates play less important roles in regulating highly abundant proteins^16,24^. Collectively, these data suggest that the increase in the content of several highly abundant proteins (including mitochondrial proteins) in human muscle might be associated, at least partially, with chaperone-dependent mechanisms that increase protein stability.

### Potential mechanisms that control the pattern of protein regulation

**Figure 4:**
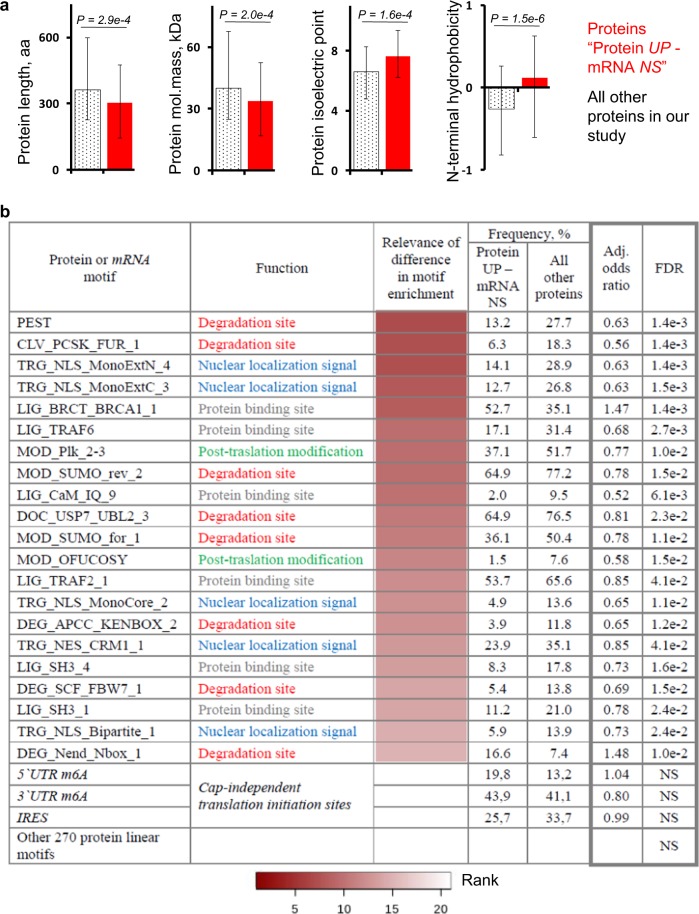
Proteins from the group “*Protein UP–mRNA NS”* demonstrate reduced enrichment of motifs associated with protein degradation. **(a**) The physicochemical properties of proteins belonging to the group “*Protein UP–mRNA NS*” and those of all other proteins detected in the present study. Data are expressed as the median and interquartile range. **(b**) Relevance of differences in motif enrichment among these protein groups (the lower the rank [dark red], the higher the relevance). Because both the motif abundance (frequency) and the adjusted odds ratio among the groups explain differences in protein regulation (or mRNA translation initiation), the relevance of differences in motif enrichment among the protein groups was calculated based on these parameters and expressed as a mean rank.

Two main mechanisms may be responsible for up-regulating proteins in the group “*Protein UP–mRNA NS*” without inducing changes in mRNA expression. First, protein-specific regulation of translation in a cap-independent manner through internal ribosome entry sites (IRESs) and N6-methyladenosine (m6A) modifications within the 5′ and 3′ UTR^25^. This might explain (at least in part) a mitochondrial-specific increase in protein synthesis induced by acute aerobic exercise^13,26,27^. Second, protein-specific regulation of stability via physicochemical properties (protein length, molecular weight, isoelectric point, and N-terminal hydrophobicity)^28^, and/or the presence/absence of regulatory motifs responsible for cleavage or ubiquitination binding^29^. We examined these parameters in proteins belonging to the group “*Protein UP–mRNA NS*” and compared them with those for all other proteins detected in the study. Proteins belonging to the group “*Protein UP–mRNA NS*” were shorter (by 21%), of lower mass (by 19%), and had a higher pI (by 1.01) and greater N-terminal hydrophobicity (by 0.37) than proteins belonging to other groups (Fig. 4a). To examine the relevance of differences in motif enrichment among groups, we calculated a mean rank for both the motif frequency and the adjusted odds ratio among the groups. We found no difference in enrichment for IRES- and m6A-associated motifs in the 5′UTR and m6A-associated motifs in the 3′UTR, which are markers of cap-independent initiation of translation (Fig. 4b). However, enrichment of 21 regulatory motifs (out of 271 investigated motifs) did differ among the group “*Protein UP–mRNA NS*” and other examined proteins. Proteins from group “*Protein UP–mRNA NS*” showed lower levels of enrichment for many motifs associated with protein degradation (e.g., PEST-, ubiquitin-, and SUMO-related motifs; Fig. 4b), suggesting reduced regulation via proteolysis, which partially explains their enhanced stability (see data in Fig. 3c).

An important limitation of the study is the lack of measurements of protein half-life and turnover. Protein synthesis and breakdown, along with mRNA content, are responsible for regulation of proteins. Synthesis rate of individual labeled proteins in human skeletal muscle was examined in a study^30^ by mass-spectrometry. Combination of this approach with RNA sequencing seems to be very promising for a deeper understanding of protein regulation in human tissues. In addition, transcriptomic and proteomic responses in human tissues show higher variability than those in cells. Therefore, increasing sample size in further studies seems to be relevant strategy for improving significance of the obtaining data.

In conclusion, the pattern of regulation of highly abundant proteins during adaptation of human skeletal muscle to regular endurance exercise depends strongly on protein function: the extracellular matrix-related protein content is regulated at the transcriptional level, whereas mitochondrial protein content is not. The increase in expression of several proteins (including mitochondrial proteins) is associated with increased protein stability via chaperone-dependent mechanisms and/or reduced proteolysis. Taken together, the data presented herein increase our understanding of the general molecular mechanisms underlying regulation of protein expression. In particular, they shed light on the molecular mechanisms underlying regulation of protein expression in human skeletal muscle subjected to repeated stress (exercise). Elucidation of these mechanisms may provide an opportunity to control the expression of specific proteins (e.g., extracellular matrix-related proteins, mitochondrial proteins) in muscle through physiological and/or pharmacological approaches.

## Methods

### Experimental model and subject details

The study was approved by the Biomedicine Ethics Committee of the Institute of Biomedical Problems of the Russian Academy of Sciences and complied with the guidelines set forth in the Declaration of Helsinki. All participants gave written informed consent to participate in the study.

Experimental model was described in our previous study^7^. Briefly, 7 untrained males (median age, 21 years [interquartile range, 21–24 years]; weight, 74 kg [72–79 kg]; body mass index, 23 kg/m^2^ [23–24 kg/m^2^]) were involved in a 2 month aerobic training program (5/week, 1 h/day) on a two-legged cycle ergometer (Ergoselect 200, Ergoline, Germany). The aerobic training program included alternating continuous (60 min, 70% LT_4_ [power at lactate threshold at 4 mmol/l]) and intermittent ([3 min, 50% LT_4_ + 2 min, 85% LT_4_] × 12) exercise on different days. 48 h after the last exercise, each subject performed a one-legged continuous knee extension exercise (∼65% W_max_) for 1 h on a modified electromagnetic ergometer (Ergometric 900 S, Ergoline, Germany). Biopsy samples were taken from the m. vastus lateralis of each leg before and after a 2 month training program (to detect baseline changes in proteome and transcriptome), as well as at 1 h and 4 h after the one-legged knee extension exercise (to detect transcriptome responses specific for contractile activity; Fig. 1a). To detect genes specifically involved in contractile activity, we examined differentially expressed genes (DEGs) between exercised and non-exercised muscle at the same time points (at 1 h and 4 h after the one-legged knee extension exercise). This design of the study provided an opportunity to eliminate effects associated with circadian regulation and systemic factors (e.g., changes in neural activity and blood metabolite content) and to identify transcriptome response specific for contractile activity. No DEGs between exercised and non-exercised muscle were found prior to exercise.

The muscle samples were taken, as described in our previous study^7^, under local anesthesia (2 mL 2% lidocaine) using a microbiopsy technique^31^, quickly blotted with gauze to remove superficial blood, frozen in liquid nitrogen, and stored at −80 °C until required.

### Protein extraction, sample preparation, and mass-spectrometry

A piece of frozen tissue (15 mg) was homogenized in 10 volumes of a lysing buffer (4% sodium dodecyl sulfate in 0.1M Tris-HCl pH 7.6, 0.1M dithiothreitol) in a 1.5 mL tube using a plastic pestle, vortexed, and incubated for 5 min at 95 °C. The samples were sonicated (2 × 10 s at 100 W) and centrifuged (5 min, 16 000 g). The supernatant (8 μL) was loaded onto the YM-30 filter (Millipore, Ireland) for alkylation and trypsinolysis (12 h, 2 μg of trypsin [Tripsin Gold, Promega, United States] in 40 μL of 0.1 M triethylammonium bicarbonate), according to the FASP method^32^. Peptides were washed off the filter by centrifugation (10 min, 14000 g); 40 μL of 0.1 M triethylammonium bicarbonate was added to the filter and the sample was centrifuged. Peptide concentrations were measured by the bicinchoninic assay; then peptides were labeled using iTRAQ 8-plex kit (Sciex, USA) according to manufacture instruction.

The mixture of labeled peptides was concentrated and subjected to reverse-phase LC fractionation at a high pH using the XBridge C18 column (250 × 4.6 mm, particle size 5 μm; Waters, Ireland) and an Agilent 1200 Series HPLC (Agilent, United States), as described elsewhere^33^. The collected fractions were dried using a vacuum concentrator and dissolved in a volume of 20 μL; the fractions were combined to obtain 10 mixed fractions and subjected to HPLC-MS/MS analysis using the HPLC Ultimate 3000 RSLCnano system (Thermo Scientific, United States).

Prior to analytical separation, the peptides were concentrated on an Accalaim μ-Precolumn (0.5 mm × 3 mm, particle size 5 μm; Thermo Scientific) in the isocratic mode at a 10 μL/min flow for 5 min in the mobile phase B (2% acetonitrile, 0.1% formic acid). The peptides were then separated on the Acclaim Pepmap C18 column (75 μm × 150 mm, particle size 2 μm; Thermo Scientific) in a gradient mode of elution. The gradient (90 min) was formed by the mobile phase A (0.1% formic acid) and B (80% acetonitrile, 0.1% formic acid) at a 0.3 μL/min flow. The column was washed with the 2% mobile phase B for 5 min; the mobile phase B concentration was increased linearly to 35% for 65 min and to 99% over 5 min. After washing the column (5 min, 99% B), the mobile phase B concentration was decreased to the initial conditions of 2% of the mobile phase B over 5 min. Mass spectrometric analysis was performed, using a Q Exactive HF Hybrid Quadrupole-Orbitrap mass spectrometer (Thermo Scientific) using the nanoelectrospray ion source in the negative mode of ionization (Thermo Scientific). The ionizing voltage was 2.1 kV. Panoramic MS spectra were acquired at the 400–1200 m/z range; fragment ions were mass scanned from m/z 100 to the upper m/z value as assigned by the charge state of the precursor ion, but not greater than 2100 m/z. All tandem MS scans were performed on ions with a charge state from z = 2+ to z = 6+. Synchronous precursor selection facilitated the simultaneous isolation of up to 20 MS2 fragment ions. The maximum ion accumulation times were set to 50 ms for precursor ions and 110 ms for fragment ions.

### RNA extraction, library construction, and sequencing

RNA was extracted from the frozen samples (~20 mg) using an RNeasy mini kit (Qiagen, Germany) with DNase I treatment (Fermentas, Lithuania), as described in our previous study^7^. The RNA concentration was measured in a fluorimeter (Qubit 3.0; Thermo Scientific) and RNA integrity was checked by capillary electrophoresis using a Bioanalyzer 2100 (Agilent, USA); all samples were at least 7 in RIN. Libraries were constructed, as described in our previous study^7^, from 300 ng of RNA using a NEB Next Ultra II RNA kit (New England Biolabs, USA). For the first stage, poly-A mRNA was isolated using magnetic particles associated with 24-dT oligonucleotides by means of NEB Next Poly(A) mRNA Magnetic Isolation Module. RNA was fragmented and reverse transcribed using 6-nucleotide random primers, followed by second strand cDNA synthesis. Adapters were ligated to the obtained cDNA, and libraries were amplified using a universal primer (the same for all libraries) and index primers. Then, libraries were cleaned using AMPure XP magnetic particles (Beckman Coulter, USA). After PCR and purification, the cDNA concentration was measured in a Qubit 3.0 fluorimeter and the length distribution of library fragments was checked by capillary electrophoresis using a Bioanalyzer 2100. The effective amplicon concentration was evaluated by real-time PCR. The libraries were diluted to 2 nM and sequenced on a HiSeq 2500 and NextSeq 500 instruments (Illumina, USA), according to the manufacturer’s instructions. Mean amount of reads was 47 [35–50] million reads per sample. Raw data has been deposited to NCBI GEO: GSE120862.

### Processing of mass-spectrometry data

The search, identification of peaks, and estimation of the intensity of the peaks of the reporter ions were carried out using the MaxQuant computational platform (1.5.7.4; Max Planck Institute of Biochemistry) at default settings for the FDR of 1%.

Processing of proteomic data and its comparison with transcriptomic data were done using the Perseus computational platform (1.6.1.2; Max Planck Institute of Biochemistry). After filtration (potential contaminants, reverse peptides, and peptides identified only by site) the ratios of intensities of the reporter ions (after training to before training) were calculated for each protein. To reduce variations related with the difference in the amount of the total proteins in samples, normalization to reference proteins was applied^34^. The ratios of intensities were normalized to the median ratio of three reference proteins showing the highest P-level (the Wilcoxon signed rank test). Then, to examine significantly changed proteins (the ratios different from 1), the Wilcoxon signed rank test with a cut off criteria for P_adj_ (Benjamini-Hochberg correction, Q-value) <0.05 was used.

### RNA sequencing data processing

Data processing was described in our previous study^7^. Briefly, the quality of RNA sequencing data for each library was assessed using FASTQC (v0.11.4), and adaptor sequences and low quality reads were trimmed using the Timmomatic tool (v0.36). Reads were aligned to the reference genome GRCh38 (with Ensembl annotation GRCh38.90) and splice junctions were detected using HISAT2 v2.1.0. HTSeq v0.6.1 was used to count the read numbers mapped to known exons of each gene (using Ensembl annotation GRCh38.90). Differential expression analysis was performed using the DESeq2 R package (analysis of paired samples) with a cut off criteria for P_adj_ (Benjamini-Hochberg correction) <0.05 and for |log_2_(Fold Change)| > log_2_(1.25). To estimate individual fold changes, the normalized read counts (the median normalization) were calculated.

As a result of processing of mass-spectrometry data, several proteins might fall in a Protein group. Therefore, for comparison of mRNA changes with protein changes, the sum of the normalized read counts for proteins in a Protein group was used.

### Functional gene ontology enrichment

Functional enrichment of protein groups in relation to all detected proteins (795 background proteins) was performed by the DAVID 6.8 using GOTERM_BP_Direct and GOTERM_CC_Direct databases. GO terms with P_adj_ < 0.05 (Fisher exact test, Benjamini correction) were regarded as significantly enriched. If several proteins fell in a Protein group, then, for the functional enrichment analysis, a gene with the greatest mRNA expression level among genes corresponding to proteins in a Protein group was chosen (*Main gene* in Dataset 1).

### Protein and mRNA motifs enrichment

To evaluate the relevance of difference in motif enrichment between different protein (or mRNA) groups we used a mean rank for both the motif abundance (frequency) and the adjusted odds ratio between the groups, because both parameters explain the difference in the regulation of proteins (or mRNA translation initiation). For each motif the odds ratio between the groups with P_adj_ < 0.05 (Fisher exact test, Benjamini-Hochberg correction) was identified. Statistically corrected (adjusted) odds ratio was calculated as the left and the right value of the 95% confidence interval for the odds ratio >1 and <1, respectively. Finally, the false discovery rate-controlling procedure (FDR < 0.05) was applied. The calculations were performed in the R environment.

*PEST motifs* in protein were predicted using the epestfind algorithm (EMBOSS v6.6.0) at threshold score +5.

Known *linear protein motifs* (270) were taken from the ELM database. The presence of a linear motif in a protein was assessed using the Sequence Manipulation Suite (Version 2).

*m6A site* were predicted in 5′UTR and near 3′UTR (+/−100 bp from the stop-codon) mRNA using the SRAMP at moderate confidence level (10% false positive rate). The UTRs and stop-codons were evaluated by the coordinates of most abundant transcripts in human (young males) skeletal muscle in the GTEx database (GTEx_Analysis_2016–01–15_v7).

*IRES* site were predicted in 5′UTR mRNA using the IRESPred.

### The physicochemical properties of proteins

The protein half-life was evaluated using data from two previous studies investigating the baseline half-life of several thousand proteins in C2C12 and mouse fibroblasts^16,23^.

Protein molecular mass, pI, and N-terminal (2–16 aa) hydrophobicity (the grand average of hydropathy [GRAVY]) were evaluated using the Sequence Manipulation Suite (Version 2).

Data are expressed as the median and interquartile range. The Mann-Whitney test was used to compare variables.

## Supplementary information

Dataset 1. Processed transcriptomic and proteomic data.

Dataset 2. Pattern of the baseline changes in protein and mRNA level and the protein half-life.
